# Partial Similarity Reveals Dynamics in Brainstem-Midbrain Networks during Trigeminal Nociception

**DOI:** 10.3390/brainsci10090603

**Published:** 2020-09-02

**Authors:** Arne May, Laura Helene Schulte, Guido Nolte, Jan Mehnert

**Affiliations:** 1 Department of Systems Neuroscience, University Medical Center Eppendorf, 20246 Hamburg, Germany; a.may@uke.de (A.M.); la.schulte@uke.de (L.H.S.); 2 Department of Neurophysiology and Pathophysiology, University Medical Center Eppendorf, 20246 Hamburg, Germany; g.nolte@uke.de

**Keywords:** pain, partial similarity, representational similarity, correlation, partial correlation, fMRI, brainstem, pain processing

## Abstract

Imaging studies help us understand the important role of brainstem and midbrain regions in human trigeminal pain processing without solving the question of how these regions actually interact. In the current study, we describe this connectivity and its dynamics during nociception with a novel analytical approach called Partial Similarity (PS). We developed PS specifically to estimate the communication between individual hubs of the network in contrast to the overall communication within that network. Partial Similarity works on trial-to-trial variance of neuronal activity acquired with functional magnetic resonance imaging. It discovers direct communication between two hubs considering the remainder of the network as confounds. A similar method to PS is Representational Similarity, which works with ordinary correlations and does not consider any external influence on the communication between two hubs. Particularly the combination of Representational Similarity and Partial Similarity analysis unravels brainstem dynamics involved in trigeminal pain using the spinal trigeminal nucleus (STN)—the first relay station of peripheral trigeminal input—as a seed region. The combination of both methods can be valuable tools in discovering the network dynamics in fMRI and an important instrument for future insight into the nature of various neurological diseases like primary headaches.

## 1. Introduction

Functional magnetic resonance imaging (fMRI) can identify hubs of neuronal networks and as such simultaneously activate areas specific to an investigated task, but allows little to no access to the connectivity within these networks and particularly no insights into their dynamic during tasks. Nevertheless, exactly these network dynamics are probably an important key to understanding the pathophysiological mechanisms of certain diseases. A good example is cycling primary headaches like migraine and cluster headaches where the network dynamics between the hypothalamus and certain brainstem nuclei change during the cycling phases of the disease, i.e., from interictal to ictal phases, leading to altered nociceptive processing [1,2,3]. However, further analysis of such dynamics is limited since causal insights into connectivity like Granger causality are not reliable for most fMRI experiments due to the poor temporal resolution [4,5,6]. The most promising alternative, dynamical causal modelling (DCM) [7], is a model with numerous limitations and constrains. The repetition time needs to be faster than 2 s and a strong a priori knowledge of all the regions of interest, their possible connectivity and how they are modulated must exist [8]. These strong prerequisites are not met by many experiments. A promising tool with fewer prerequisites are psychophysiological interactions (PPI) [9], yet it still depends on a minimum of two contrasting conditions. Both DCM and PPI work on the amplitude of the BOLD signal. Although the height of the amplitude is a strong indicator of the level of regional activity, a conventional connectivity analysis only using the absolute height as a marker may miss a functional connection since two regions can be highly connected but exhibit little stimulus-related activity since they can process more than just one input simultaneously.

Correlation analysis offers an alternative to inform about connectivity between spatially separated regions. One of the first approaches was to extract regional time courses and to calculate their correlation, as was done in the first resting-state studies [10]. However, this approach has been shown to be susceptible to movement artifacts, cardiovascular changes, and high homogeneity within individual MR-slices [11,12], yielding spurious correlations. A question arising when using time-course correlation for task-dependent data is how many time points should be used when considering the correlation of time courses during a given task. Too many time points would mostly detect connectivity at baseline, while too few points may underestimate the actual connectivity. An additional question is how to handle dynamic stimuli, i.e., a stimulus that appears sometimes in the middle and sometimes at the end of a MR-volume (TR).

One way to overcome these limitations is to use single-trial activity patterns estimated by modeling each trial individually in a GLM [13,14,15]. Such data are more robust to analyze since they are already corrected for motion, slice timing, and overlapping hemodynamics. This kind of analysis is also referred to as beta time series correlations [13,16,17,18,19]. Trial-to-trial variability is, amongst others, based on changes of input strength (i.e., slight changes of stimulus), random fluctuations in attention and variations in endogenous (e.g., pre-stimulus) brain activity that modulates stimulus-evoked responses [13,20]. Such random fluctuations can inform communication between a seed area and all functionally connected cortical (and subcortical) areas; using a focused analysis allows to investigate network dynamics in fMRI datasets [15]. This more abstract level of data correlation is framed as Ordinary Similarity or classical Representational Similarity Analysis [21,22] and has been shown to be reliable even for fast event-related designs and accordingly overlapping hemodynamics [13,14,16,17,18,19]. Nevertheless, even simple simulations show that ordinary correlation analysis can lead to false positive as well as false negative connectivity results (see Supplement, cases 3 and 4). The connectivity between two hubs of a given network can be overestimated due to the existence of a strong global network signal. This means on a neuronal level, that the connectivity can be trivial when the whole network shows the same variation of activity. The same holds true when a third hub influences both hubs of interest. An example of underestimated connectivity would be that a third hub interferes with both hubs of interest, such that it modulates the first hub positively but the other negatively (Supplement 1, case 4). This case can also be seen as an example of two highly connected regions which process more than just one input and therefore show little stimulus-correlated activity as it represents only one part of the ongoing processing. To overcome this problem, partial correlation analysis could be used as complementary where the direct correlation between two hubs is controlled for other influences. This has been shown for the motor cortex by Marrelec and colleagues [23] using raw time courses of fMRI data as input for their analysis and is in similar ways also used to analyze resting state data e.g., [24,25]. Considering the critique on correlations of time courses, we extended the Ordinary Similarity approach to Partial Similarity analysis where ordinary correlation is replaced by partial correlation to discover more specifically the direct communication between two individual regions. Using Partial Similarity analysis the communication between two hubs is corrected for a third hub, multiple other hubs, or even the rest of all network activity (including noise within the network) by subtracting their activity beforehand. Partial Similarity should be seen as being complementary to Ordinary Similarity; while Ordinary Similarity reveals a summary on the connectivity between two hubs which includes global as well as direct connectivity, Partial Similarity focuses only on the direct connectivity between them.

We tested our approach using simulations presented in the Supplementary Document 2 and in brainstem-optimized fMRI data where 29 subjects underwent 15 trials of strong nociceptive trigeminal input into the left nostril as a painful condition and air puffs in the control condition. This leads to well-known activation of the trigeminal system [3,26,27], with the spinal trigeminal nucleus being the first relay station of the central nervous system for incoming trigeminal input [28]. Combining Representational Similarity with Partial Similarity, we were able to more reliably describe the trigeminal nociceptive network and additionally unravel direct connectivity between individual hubs of this network which may otherwise be hidden.

## 2. Materials and Methods

### 2.1. Simulations

To prove the translation of using partial correlations for beta-time series analysis, we ran simulations to evaluate the complementary information revealed by Ordinary and Partial Similarity. In summary, we simulated several amounts of global network signals mixed into 100,000 voxel with 435 time points, i.e., betas. The simulations include: (i) no direct connectivity between a seed and a target voxel, (ii) several levels of direct connectivity between a seed and a target voxel, and (iii) a situation where the seed and the target voxel are influenced by one or more regions of a global network signal which has excitatory influence on the seed but inhibitory influence on the target voxel. We present these simulations in the Supplementary Document 2 entitled “Simulations on the effect of a global network signal on Ordinary and Partial Similarity”.

### 2.2. Subjects and Experimental Design

Twenty-nine healthy volunteers participated in our experiment on trigeminal nociception which has been repeatedly shown to prompt robust results (see Schulte et al. [29,30,31]). The current data was partly already published [29,30,31]. In short, participants received 15 trials of gaseous ammonia as nociceptive trigeminal input and 15 air puffs as a control condition. Each trial was rated for intensity and unpleasantness. The standardized experiment also included 15 trials of rose odor and 15 trials of visual stimulation not analyzed for this work. Written informed consent was obtained from all participants and the study was conducted according to the Declaration of Helsinki and approved by the Ethics Committee in Hamburg, Germany (PV 4522). The datasets generated and analyzed during the current study are not publicly available due to national data protection acts. Data are available from the corresponding author upon reasonable request.

### 2.3. Image Acquisition

MR-Images were collected on a 3T scanner (TRIO, Siemens, Munich, Germany) using a 32-channel head coil. Functional images were acquired with high-resolution EPI optimized for the human brainstem (38 axial slices, 1.25 × 1.25 × 2.5 mm^3^, TR 2.61 s, TE 27 ms, FOV 216 mm^2^, GRAPPA accelerated, 2 saturation pulses) [29]. The measured volume was restricted to the brainstem including the Thalamus and parts of the Cingulate Gyrus as the top border and the foramen magnum as the bottom border. The cerebellum was only partly within the measured volume. High-resolution structural images were obtained using an MPRAGE sequence with 1 mm^3^ isotropic resolution [29].

### 2.4. Preprocessing

Functional and structural images were denoised using a spatially adaptive non-local means filter [32] as implemented in the CAT12 toolbox (http://www.neuro.uni-jena.de/cat/). Functional images were then realigned and slice time corrected using SPM12 (Wellcome Trust Center for Neuroimaging, London, UK). The average EPI was further co-registered to the participants’ structural image. To realize our analytical approach, we calculated one General Linear Model (GLM) where each painful and each control trial was individually modeled with an HRF and included them as regressors of interest in a trial-by-trial GLM [13,15], while visual and odor condition were included as single, condition-wise regressors. Furthermore, we included models of button presses, the 6 movement regressors obtained in the realignment step within the preprocessing, and the discrete cosines (DCT), used in the standard SPM analyses as a high pass filter (128 Hz), in the GLM analysis. For physiological noise correction we additionally included 18 to 20 regressors extracted from the subjects’ breathing and pulse signals with the approach described by Deckers and colleagues [33]. Resulting trial-specific beta-images were then normalized to MNI space with an isotropic voxel size of 2 mm^3^ using the segmentation of the participants’ structural image as implemented in SPM12 and smoothed by a 2 mm^3^ isotropic Gaussian kernel and are the basis of all further analyses.

We then compared the trial-by-trial GLM results [13] to previously published results on condition-wise GLMs [2,29,34], calculating a contrast image with all trials on the subject level which entered a group level one-tailed t-test with an uncorrected threshold of *p* < 0.0001.

**Figure 1:**
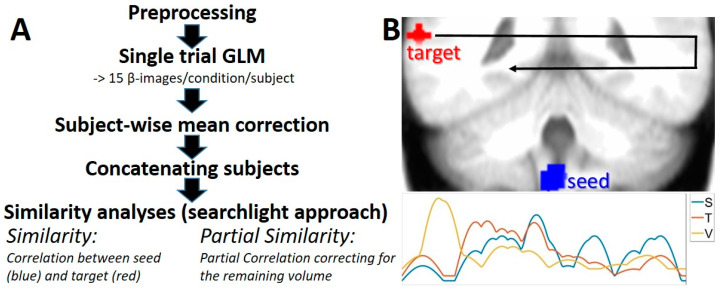
Sketch of the analyzing path used for the current study. (**A**) Similarity and Partial Similarity use concatenated trial-by-trial beta-images from the GLM analyses. (**B**) The searchlight approach used for the analysis involves the STN as seed (S, blue) whereas the target (T, red) represents a sphere wandering (represented by the black arrow) through the volume in a searchlight approach. Any signal that is detected in all volumes (such as movement artefacts) is used for correction in the Partial Similarity analysis. The plot bottom right shows three possible time courses for seed, target and general signal shared by the whole volume (V).

Trial-by-trial beta-images were further subject-wise subtracted by their mean and concatenated across subjects (Figure 1). We used a mask for gray and white matter gained from segmenting the average of the spatially-adaptive non-local mean corrected and normalized structural images. Furthermore, all voxels with zero variance were rejected, leaving about 100,000 (precisely 96,973) voxels for the proposed analysis.

### 2.5. Plausibility Checks

To gain first insights into the meaning of the variance of trial-by-trial activity we ran a Principal Component Analysis (PCA) on the preprocessed, concatenated and standardized data. A PCA finds uncorrelated components of the data, which are sorted by their explained variance, and is used to reduce high dimensional data. The first component is therefore the most meaningful compartment of the data. Its weights explain which voxels contribute to which extent to the component and give important insights into the data quality and hint its meaning [35,36,37].

### 2.6. Formulation of Similarity and Partial Similarity

As a measure of connectivity between a seed area *x* and a target area *y*, we calculated Spearman correlation coefficients between their spatial means referred to as (representational) Similarity [21]. Spearman correlation can be understood as a special case of classical Pearson’s correlation using the rank *rg* of the sorted entries of *x* and *y* and can be formulated as

(1) $$CORR\left(x,y\right)=\frac{COV\left(rg_{x},rg_{y}\right)}{s_{rg_{x}}s_{rg_{y}}},$$

where *COV* is the covariance matrix and *s* is the standard deviation.

We further introduce Partial Similarity, which is also based on the Spearman correlation but corrects for matrix *C* by subtracting a weighted, linear product of *C* before calculating the correlation coefficient, i.e., performs a partial correlation. This can be formulated by

(2) $$PARTIALCORR\left(x,y\right)=CORR\left(x-w_{x}C,y-w_{y}C\right),$$

where *x* and *y* are again the spatially averaged beta-estimates of seed and target and *w* weights the vectors in *C*. In *C* we find the first components from the transform along the variance of the standardized beta-images of V (Volume of No Interest) by a PCA. This transformation is used for data reduction which becomes necessary because the number of concatenated beta-images limits the number of components, which can be used as controlling variables within the partial correlation, i.e., more controlling variables than the number of elements in *x* (and thereby *y*) are not possible. *C* is formulated as

(3) C=bV,

where *V* are the standardized entries of all voxels with the Volume of No Interest (sketched in Figure 1). The first component *C_1_* of *V* is constructed by finding its weight *b_1_* according to

(4) $$b_{1}=arg\;\underset{∥b∥=1}{\max}\left\{\underset{i}{\sum }{\left(V_{\left(i\right)}b\right)}^{2}\right\}\;$$

and the further components *C_i_* iteratively by finding their weights *b_i_* using an altered $\tilde{V}$ in Formula 4 with the already calculated components subtracted from *V*.

Finally, the weight *w* used in Formula 2 can then be estimated with simple linear regression for *x* and *y*, respectively, using

(5) $$w_{x}=arg\;\underset{w}{\min}\left\{\overset{N}{\underset{i=1}{\sum }}{\left(x_{i}-\langle w_{x},c_{i}\rangle \;\right)}^{2}\right\}.$$

### 2.7. Application of Similarity and Partial Similarity

In the presented analyses we chose the left spinal trigeminal nucleus (STN) as a seed region as it is the first relay station in the central nervous system for trigeminal nociceptive input [28]. As the center of the seed, we used MNI coordinates (−4, −46, −53) from a published independent sample [34] and constructed a sphere around it with a radius of 8 mm. We further excluded the cerebellum from this sphere by masking it with the template provided by the SUIT toolbox for SPM 12 [38] normalized to MNI space.

The target area is constructed as a sphere with a radius of 8 mm (like the seed) and wanders around the entire volume using a searchlight approach [39] such that each voxel is the center of the target in exactly one iteration. The spatial average is used for calculating Similarity and Partial Similarity.

To perform Partial Similarity, the activity of the volume of no interest is first reduced along the variance into its first 15 components explaining a minimum of 30% of the variance (100 randomly chosen voxel were used for this estimation) by PCA as described above. Due to the high homogeneity between nearby areas, we defined an additional space with a radius of 15 mm around seed and target regions which were excluded from the analyses to prevent partial correlation coefficient of only −1 and 1. The proposed analyses are sketched in Figure 1.

### 2.8. Statistics

The *p*-values stemming from the calculated Spearman correlations and partial correlations are corrected for multiple comparison using the Benjamini–Hochberg [40] and the Benjamini—Yekutieli [41,42] procedure for controlling the false discovery rate (FDR) of a family of hypothesis tests [43]. All reported statistical tests are one-tailed.

## 3. Results

### 3.1. Simulations

The first simulation shows that a global network signal induces a strong connectivity between seed and target as measured by Ordinary Similarity analysis which can reach correlation values close to 1 when the influence of the global network signal is extreme (Figure S2). The Partial Similarity analysis shows, as expected, that there is no direct connectivity between seed and target (Figure S2). As the maximally measured correlation coefficients in the actual data reaches levels of around r = 0.2 and the explained variance of the controlling variables is around 30%, the global network signal might have an influence of up to 60% in our real fMRI beta time series data.

The second Simulation shows the complementary information of Ordinary and Partial Similarity: While the Ordinary Similarity reveals connectivity induced either by the global network signal or direct connectivity or a mixture of both, Partial Similarity only reveals the direct connectivity component.

Simulation 3 tests to mislead Ordinary Similarity by adding a global network signal to the seed but subtracting it from the target. Here, Ordinary Similarity can reveal even negative correlations or near-zero correlations when the magnitude of the global signal and the direct connectivity is similar. Details of the simulations can be found in the Supplementary Document 2.

### 3.2. Plausibility

**Figure 2:**
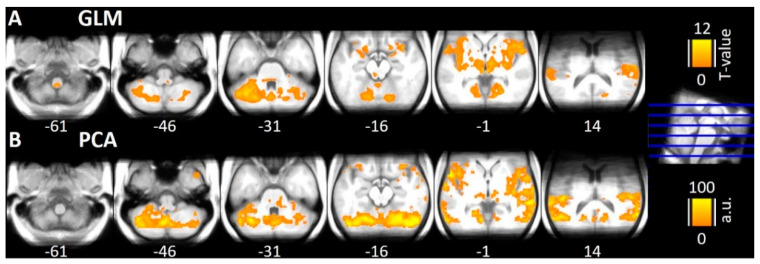
(**A**) Results of the trial-by-trial GLM analyses, which provides the basis for the proposed Similarity and Partial Similarity analysis. For visual inspection, results are shown using an uncorrected statistical threshold of *p* < 0.0001. The results confirm previously published condition-wise calculated GLMs [29,30,34]. (**B**) Normalized weights of the 1st component of the Principal Component Analysis (PCA), which explains nearly 10% of the concatenated beta-images resulting from the GLM analysis shown in the top row. Most of the known hubs of trigeminal nociception like STN, PAG, rostral Pons, Thalamus, and Cerebellum are involved. These unsupervised results underline the potential of analyzing trial-by-trial variations.

Trial-by-trial GLM analysis confirmed previously published results [2,26,27,29,34,44,45,46], i.e., significant activation in left STN, rostral Pons, Cerebellum, periaqueductal gray (PAG), Thalamus, and bilateral Insula (Figure 2A). Due to the restricted measured volume, no activation could be shown in other areas. The first component of the PCA on the concatenated trial-by-trial beta-images from the GLM explains 9.85% of the overall variance (voxel × trials) and reveals a plausible network within the brainstem during trigeminal nociception. The network includes previous reported hubs of trigeminal nociception in the brainstem and midbrain such as the STN, rostral Pons, Cerebellum, PAG, bilateral Thalamus and bilateral Insula (Figure 2B). This unsupervised analysis underlines the potential of analyzing trial-by-trial variance.

### 3.3. Similarity and Partial Similarity

**Figure 3:**
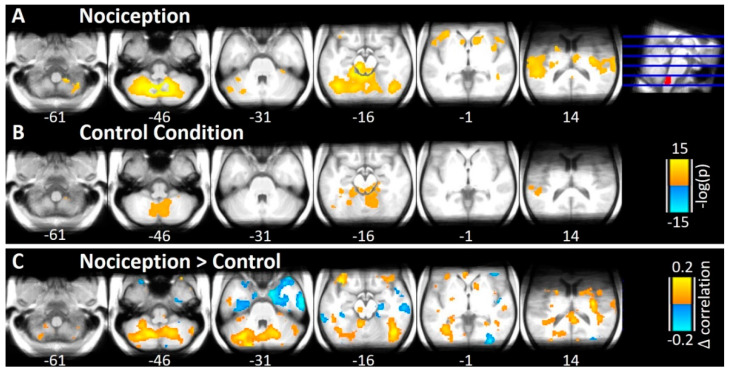
Results of the Similarity analysis. (**A**) Log-transformed significance of correlations (Spearman) in trial-to-trial variability between the left STN (marked in red at the top right corner) and cerebellum, brainstem, and midbrain during nociception at a statistical threshold of *p* < 0.0001 (FDR-adjusted) and a minimum cluster extent of 5 voxel. (**B**) The same results for the control condition. (**C**) Difference of the correlation coefficients between the two conditions (nociception > control). Positive findings signify higher correlation during nociception than during air puffs (marked in orange) whereas negative correlation is depicted in blue.

Similarity analysis from the STN ROI as a seed reveals three clusters with positive correlations and 16 clusters with negative correlations at a statistical threshold of FDR-adjusted *p* < 0.05, where the most extensive cluster contains more than 54% of all considered voxel, especially most of the midbrain. To pinpoint more specific regions, we increased the statistical threshold to an FDR-adjusted *p*-value of *p* < 0.0001, revealing nine clusters with similar trial-to-trial activity changes as the STN in the nociceptive condition (Figure 3A) with a minimum cluster extent of 5 voxel (40 mL). The clusters mostly include cortical and subcortical structures already shown to be involved in trigeminal nociception like bilateral Cerebellum, bilateral PAG, bilateral Insula, bilateral Operculum, right Striatum (Caudate, Putamen), and bilateral Thalamus and furthermore the right Cingulate Gyrus (Table 1).

The control condition with air puffs also showed highly correlated midbrain areas at a threshold of FDR-adjusted *p* < 0.05. Regarding nociception, we therefore increased the threshold to *p* < 0.0001 (Figure 3B, Table 1) and identified seven clusters correlated with the left STN seed region in their trial-to-trial variance. These clusters included the bilateral Cerebellum, bilateral PAG, the left inferior Temporal Gyrus, left Fusiform Gyrus, and the right Planum Polare (Table 1).

**Figure 4:**
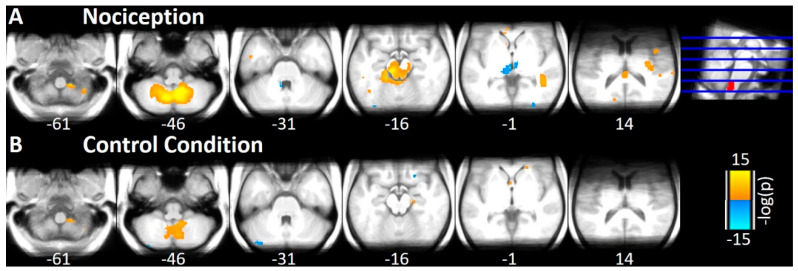
Results of the Partial Similarity analysis. (**A**) Log-transformed significance of partial correlations (Spearman) in trial-to-trial variability between the left STN (marked in red at the top right corner) and cerebellum, brainstem, and midbrain during nociception at a statistical threshold of *p* < 0.05 (FDR-adjusted) and a minimum cluster extent of 20 voxel. The correlation between the STN and the individual voxel is controlled for general signals from the rest of the volume. (**B**) The same results for the control condition. Positive partial correlations are marked in orange, negative partial correlations are marked in blue.

**Figure 5:**
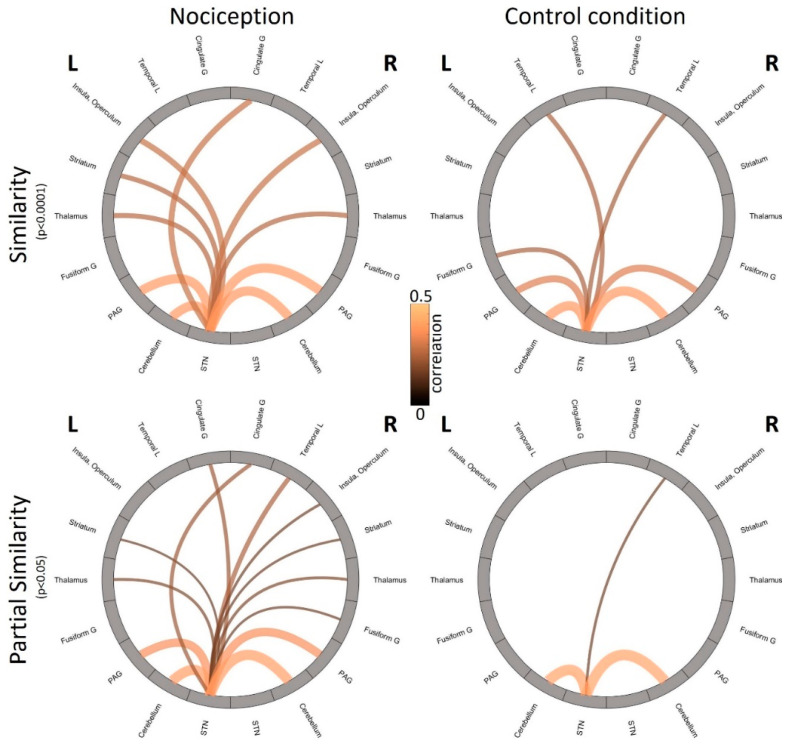
Plot of the interactions revealed by Similarity (**top**) and Partial Similarity (**bottom**) of the left STN to brainstem, cerebellum, midbrain, and cortical areas in the field of view during trigeminal nociception (**left**) and control condition (air puffs, **right**). Each circle shows connections to the left hemisphere (**L**) on the left hand side of the circle and connections to the right hemisphere (**R**) on the right hand side the circle. Lines mark connections. Color and thickness of the lines signify the strength of the connection (correlation coefficient or partial correlation coefficient). G = Gyrus, L = Lobe. STN = Spinal trigeminal nucleus, PAG = Periaqueductal gray matter. Due to the restricted volume, no connectivity can be shown to other cortical areas.

Partial Similarity analysis of the nociceptive condition revealed 10 clusters with positive correlation and 3 negative correlation to STN (Figure 4A) at an FDR-adjusted threshold of *p* < 0.05 and a minimum cluster extent of 20 voxel (160 mL). The included regions are bilateral Cerebellum, PAG, and Cingulate Gyrus, parts of the temporal lobe (right Planum Temporale, right Middle Temporal Gyrus, left Inferior Temporal Gyrus), bilateral Striatum (Caudate), and with a negative correlation the right Fusiform Gyrus and both sides of the Thalamus (Figure 5, Table 2). Most of the regions are well known to process painful trigeminal input.

In the control condition, we observed four clusters with positive correlations and one cluster with negative correlations to the STN (Figure 4B, Table 2), namely bilateral Cerebellum and the Temporal Pole (Figure 5).

To gain an overview of the reported results, we sketched the revealed connectivities and their strength from the Similarity and Partial Similarity analyses in Figure 5. While both connectivity analyses show strong connectivities to Cerebellum and PAG during nociception and reveal the direct connectivity from STN to (contralateral) Thalamus, the strong contralateral crossing to higher cortical areas (Temporal lobe, Insula, Fusiform Gyrus) is only revealed using Partial Similarity analysis. In the control condition, the Similarity analyses suggest a network between left STN and bilateral Cerebellum and bilateral PAG. The latter could not be identified in the Partial Similarity analysis.

## 4. Discussion

Uncovering cortical and subcortical connectivity in human fMRI data is a challenging field as to date only a few methods exist. While commonly used DCM and PPI both work on raw time courses, they depend on the height of BOLD amplitude, and are further hindered by a long list of assumptions and constraints, ordinary correlation analysis may lead mostly to an overestimation of results. Here we introduce a new way to delineate direct (dynamic) network connectivity complementary to ordinary correlation analyses of beta time series: Partial Similarity.

We conceptually proved that Partial Similarity and its counterpart, (based on ordinary correlations) Ordinary Similarity, reliably reveal dynamic connectivity of brainstem networks known from preclinical studies. As a physiological model, we specifically chose the connectivity of the spinal trigeminal nucleus, which is the first central hub of all incoming peripheral trigemino-nociceptive input into the central nervous system (CNS). Studies on mammals suggest direct anatomical connections to the cerebellum [47,48,49], Thalamus [50], and PAG [50,51,52], which are known to be involved in mechanical and nociceptive input [49,51,53,54]. Additional to these well-known hubs, Similarity further reveals strong connectivity to the ipsilateral Insula and Operculum, which was not observed with Partial Similarity. Partial Similarity on the other hand emphasizes connectivity to contralateral regions like the striatum, Fusiform Gyrus, Temporal lobe, and ipsilateral Cingulate Gyrus. These differences stem from the nature of the two measures, which we demonstrated in our simulations: while Similarity shows overall communication between two regions, Partial Similarity focuses on direct communication between two hubs, diminishing trivial and induced correlations, but uncovering correlations hidden by other sources or a global network signal. Both analyzing techniques contain complementary information (direct communication and overall communication), which should be exploited in future research by locating the origin of these differences. We suggest that imaging studies, which investigate relationships in and between neuronal networks, will strongly profit from the combination of both analytical tools. This is particularly true for research investigating cycling pathologies like migraine and cluster headaches [55], where changes in network dynamics [1] follow the current stage of the patients’ disease [2,3,56]. While we concentrate on the first hub of the trigeminal driven network in the CNS, further research will reveal the dynamical connectivity of all hubs in the network and may identify cortical and subcortical structures by combining both analytical tools, which would be missed if only one of these tools were used. Moreover, given the open-ended search feature of the proposed analyses, we aim to identify connectivity and its dynamics and hubs, which is not yet within the focus of current research. Further work will have to include the translation of Partial Similarity to other fields of Representational Similarity applications [21], where trivial correlations currently might hinder a deeper insight into direct relations, for example in the field of complementing fMRI and electroencephalography (EEG) [57]. Partial Similarity and Similarity analyses as established in the current work might thus be an important tool to discover neuronal network dynamics in fMRI and thus grant more detailed insights into the pathophysiological mechanisms underlying neurological diseases, including but not limited to primary headache disorders.

## 5. Conclusions

We have shown with both simulated and real task-dependent fMRI data that the Partial Similarity method presented here can represent direct connectivity between two hubs. Specifically, Partial Similarity can unravel brainstem dynamics involved in trigeminal pain. It thereby gains complementary information to Ordinary (also called Representational) Similarity, which reveals a summary of global and direct connectivity. The combination of both methods can be valuable tools in discovering network dynamics in fMRI and an important instrument for future insight into the nature of pathological pain processing and various other neurological diseases.

## Figures and Tables

**Table 1:** Results for the Similarity analysis during trigeminal nociception (gaseous ammonia) and control condition (air puffs) at a statistical threshold of FDR-adjusted *p* < 0.0001 and a minimum cluster extent of 5 voxel (40 mL). Coordinates are presented in MNI space.

Region	x	y	z	Cluster Size (vx)	Correlation (Peak)	Mean Correlation	*p*-Value (Peak)	FDR-Adjusted *p*-Value (Peak)
*Nociception*								
r Cingulate G	4	−44	23	123	0.29	0.23	6 × 10^−10^	2 × 10^−8^
r Insula, Operculum	32	−16	15	872	0.29	0.23	2 × 10^−10^	1 × 10^−8^
l Insula, Operculum	−50	−34	13	1512	0.29	0.23	3 × 10^−10^	1 × 10^−8^
b Thalamus	−2	0	7	435	0.26	0.21	2 × 10^−8^	5 × 10^−7^
r Caudate, Putamen	18	16	3	329	0.26	0.22	2 × 10^−8^	5 × 10^−7^
r Operculum	48	14	1	71	0.22	0.21	1 × 10^−6^	2 × 10^−5^
r Insula	42	0	−5	116	0.26	0.22	2 × 10^−8^	5 × 10^−7^
l Insula, Operculum	−26	26	−11	622	0.27	0.22	6 × 10^−9^	2 × 10^−7^
b Cerebellum, PAG	−20	−56	−49	9358	0.38	0.25	8 × 10^−19^	1 × 10^−16^
*Control condition*								
b Cerebellum	−58	−16	7	378	0.25	0.23	6 × 10^−8^	6 × 10^−6^
r Planum Polare	60	−2	5	35	0.24	0.22	3 × 10^−7^	2 × 10^−5^
l Inferior Temporal G	−48	−58	−11	72	0.25	0.23	5 × 10^−8^	5 × 10^−6^
l Fusiform G	−36	−36	−17	44	0.25	0.23	4 × 10^−8^	4 × 10^−6^
b Cerebellum, PAG	−14	−34	−21	1092	0.32	0.24	6 × 10^−12^	3 × 10^−9^
b Cerebellum	16	−44	−49	1518	0.38	0.26	5 × 10^−18^	8 × 10^−15^
l Cerebellum	−38	−52	−53	6	0.22	0.22	2 × 10^−6^	7 × 10^−5^

b = bilateral, l = left, r = right, G = Gyrus, PAG = Periaqueductal Grey.

**Table 2:** Results for the Partial Similarity analysis during trigeminal nociception (gaseous ammonia) and control condition (air puffs) at a statistical threshold of FDR-adjusted *p* < 0.05 and a minimum cluster extent of 20 voxel (160 mL). Coordinates are presented in MNI space.

Region	x	y	z	Cluster Size (vx)	Correlation (Peak)	Mean Correlation	*p*-Value (Peak)	FDR-Adjusted *p*-Value (Peak)
*Nociception*								
b Cingulate G	4	−44	23	264	0.22	0.16	3 × 10^−6^	1 × 10^−4^
r Planum Temporale	34	−22	21	299	0.25	0.17	8 × 10^−8^	7 × 10^−6^
r Insula, Operculum	44	2	17	25	0.17	0.15	2 × 10^−4^	5 × 10^−3^
r Caudate	10	18	5	53	0.16	0.14	5 × 10^−4^	1 × 10^−2^
l Caudate	−8	10	1	41	0.15	0.14	1 × 10^−3^	3 × 10^−2^
l Thalamus	−2	−20	1	115	−0.18	−0.15	9 × 10^−5^	3 × 10^−3^
r Thalamus	4	−22	1	28	−0.18	−0.15	9 × 10^−5^	3 × 10^−3^
r Middle Temporal G	44	−42	−1	266	0.21	0.16	7 × 10^−6^	4 × 10^−4^
r Fusiform G	30	−76	−3	30	−0.16	−0.14	5 × 10^−4^	1 × 10^−2^
l Inferior Temporal G	−50	−38	−13	50	0.17	0.15	2 × 10^−4^	6 × 10^−3^
b PAG	−10	−26	−21	1262	0.36	0.20	9 × 10^−15^	1 × 10^−11^
r Fusiform G	44	−40	−25	25	0.16	0.14	4 × 10^−4^	1 × 10^−2^
b Cerebellum	16	−42	−53	2929	0.47	0.22	8 × 10^−25^	6 × 10^−20^
*Control condition*								
r Cerebellum	38	−60	−57	61	0.20	0.17	2 × 10^−5^	3 × 10^−3^
b Cerebellum	12	−48	−51	911	0.41	0.22	9 × 10^−19^	9 × 10^−14^
r Temporal Pole	26	0	−37	25	0.17	0.16	2 × 10^−4^	2 × 10^−2^
l Cerebellum	−42	−78	−25	98	−0.19	−0.17	4 × 10^−5^	7 × 10^−3^

b = bilateral, l = left, r = right, G = Gyrus, PAG = Periaqueductal Grey.

## Supplementary Materials

The following are available online at https://www.mdpi.com/2076-3425/10/9/603/s1: The Supplementary Materials include detailed descriptions and results of the cases and simulations referred to in the article. It consists of: Document 1: “Cases of Ordinary and Partial Similarity“ including: Pseudocode P1: Code used to construct the 4 sample cases for ordinary and partial correlations; Figure S1: Simulated cases where ordinary and partial correlations show the same (Case 1 and 2) and divergent results (Case 3 and 4). Document 2: “Simulations on the effect of a global network signal on Ordinary and Partial Similarity “ including: Pseudocode P2: Code used to conduct the simulations on the effect of a global network signal on (non-existing) direct connectivity between seed and target revealed by ordinary and partial correlations.; Pseudocode P3: Code used to conduct the simulations on the effect of a global network signal on several levels of direct connectivity between seed and target revealed by Ordinary and Partial Similarity; Figure S2: Results of the simulation of the Ordinary and Partial Similarity analysis of the simulated beta time series for 101 steps of 10% increment of global network influence while no direct connectivity between seed and target is simulated; Figure S3: Results of the simulation of the Ordinary and Partial Similarity analysis of the simulated beta time series for 11 steps of 10% increment of global network influence while direct connectivity between seed and target is also simulated for 11 steps of 10% increment; Figure S4: Results of the simulation of the Ordinary and Partial Similarity analysis of the simulated beta time series for 11 steps of 10% increment of global network influence. Here the global network signal has an additive effect on the seed and a negative effect on the target voxel.

## Abbreviation

Abbreviation	Definition
b	Bilateral
BOLD	Blood Oxygenation Level Dependent
CNS	Central Nervous System
DCM	Dynamic Causal Modelling
DCT	Discrete Cosine Transform
EEG	Electroencephalography
EPI	Echo-Planar Image
FDR	False-Discovery Rate
fMRI	functional Magnetic Resonance Imaging
FOV	Field of View
G	Gyrus
GLM	General Linear Model
GRAPPA	Generalized Autocalibrating Partial Parallel Acquisition
l	Left
MNI	Montreal Neurological Institute
MPRAGE	Magnetization Prepared Rapid Gradient Echo
MR	Magnetic Resonance
PAG	Periaqueductal Gray
PCA	Principal Component Analysis
PPI	Psycho-Physiological Interactions
PS	Partial Similarity
r	Right
ROI	Region of Interest
STN	Spinal Trigeminal Nucleus
TE	Echo Time
TR	Repetition Time
