# Supplementary material for: Partial Similarity Reveals Dynamics in Brainstem-Midbrain Networks during Trigeminal Nociception
Source: Brain Sci. 2020 Sep 2;10(9):603. doi: 10.3390/brainsci10090603 (PMC7563756; doi:10.3390/brainsci10090603)
Supplement: Supplementary file 1 [file brainsci-10-00603-s001.pdf]

Supplementary Materials: Partial Similarity Reveals Dynamics in Brainstem-Midbrain Networks during Trigeminal Nociception

Arne May, Laura Helene Schulte, Guido Nolte and Jan Mehnert

Document 1: "Cases of Ordinary and Partial Similarity"

Using ordinary correlation may over- or underestimate the actual direct connectivity between a seed and a target region. The true direct connectivity might be discovered using partial correlations, which correct for influences of other regions (here termed "volumes of no interest"). Constructing such cases is simple (see Pseudocode 1) and might play an important role in connectivity analysis of neuroimaging data. This is the case when there is a global network signal, i.e. trivial correlations induced by all hubs of a network reacting simultaneously in the same way, or when a third region influences both, seed and target(Case 3 in Figure S1).

```
x1=0:0.01:π*4;
x2=x1*1.5;
x3=x1*2.3;

v=cos(x1);
Case 1: no correlation between S and T
s=cos(x2);
t=cos(x3)+v;
Case 2: correlation between S and T
s=cos(x2);
t=cos(x2)*0.7;
Case 3: correlation between S and T induced by V
s=cos(x2)+v;
t=cos(x3)+v;
Case 4: hidden communication between S and T: V pushes S but inhibits T
s=cos(x2)+v;
t=cos(x2)-v;
```

Pseudocode P1. Code used to construct the 4 sample cases for ordinary and partial correlations. S = seed, T = target, V = Volume of No Interest

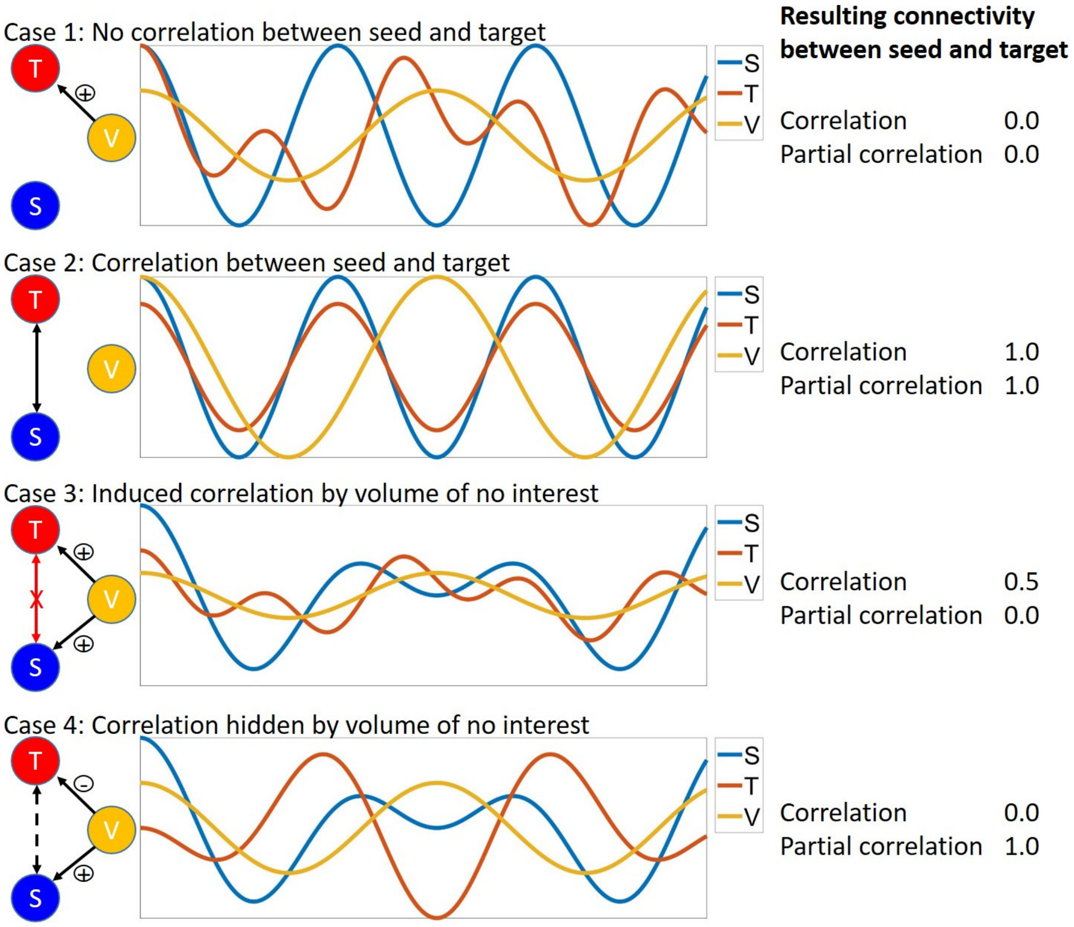

Figure S1. Simulated cases where ordinary and partial correlations show the same (Case 1 and 2) and divergent results (Case 3 and 4). Case 1 shows neither correlations nor partial correlations between seed (S) and target (T). The target is nevertheless influence by the Volume of No Interest (V). In Case 2, seed and target are correlated but though V has no influence on neither, ordinary as well as partial correlation are equally high. In Case 3 ("induced correlation") the interaction between the seed and target is overestimated as they are not directly interacting but are both influenced by V. While ordinary correlation shows a strong connectivity (c = 0.5), the influence of V is corrected for in the partial correlation which therefore becomes zero. Case 4 ("hidden correlation") gives an example of underestimated connectivity where the relation between seed and target is overwritten by an excitatory influence of V on the seed but an inhibitory influence on the target. Latter connectivity can also be discovered by partial correlation but not using common correlation.

Document 2: "Simulations on the effect of a global network signal on Ordinary and Partial Similarity"

The representation of the communication between a seed and a target region analyzed by Ordinary Similarity, i.e. classical Representational Similarity, analysis on beta time series might be effected by an global network signal, i.e. trivial correlations induced by all hubs of a network reacting simultaneously in the same way or a third region that influences both, seed and target. One way to estimate the direct communication between two regions is the use of Partial Similarity, which corrects for the global network signal. To simulate the effect of a global network signal on the connectivity estimated by Ordinary and Partial Similarity we simulated 100,000 voxel and 435 trials, i.e. time points of a beta time series as in the real data. Each of the voxel contains a time course of independent random Gaussian noise ("white noise").

Simulation 1

The global network signal is simulated by a time course of random Gaussian noise. In the first simulation (Pseudocode P2 and Figure S2) there is no direct connectivity but the global network signal is mixed with the seed and target voxels' (here voxel 0 and voxel 1) time course using 101 ascending proportions with steps of 10%. For Ordinary Similarity Analysis we now calculate the Spearman correlation of seed and target while for the Partial Similarity we run a Principal Component Analysis (PCA) on the voxel of no interest, i.e. the 99,998 voxel that are neither seed nor target. The PCA gives us uncorrelated components of the voxel of no interest sorted along their explained variance. From these we take the first 15 component as controlling variables for the Partial Spearman correlations used for the Partial Similarity analysis because they should fetch the global network signal. Furthermore, we calculate the percent of explained variance by these 15 components.

```
create a matrix with Gaussian noise in 100,000 voxel and 435 trials i.e. the beta time series
x_raw=random(10000,435);
create Gaussian noise used to induce correlations by the global network signal
g=random(1,435);
repeat for 101 mixing levels
for i=0 until 100
    add global network signal with steps of 10% to all voxels
    x=x_raw+i/10*g;
    calculate ordinary Spearman correlation between 1st and 2nd voxel, i.e. seed and target
    similarity(i)=spearmanCorr(x(0,:),x(1,:))
    calculate PCA for the remaining voxels excluding seed and target
    [b, C] = PCA(x(2:end,:))
    calculate partial Spearman correlation  controlling for 15 PCs
    partialSimilarity(i)=partialSpearmanCorr(x(0,:),x(1,:),C(0:14,:))
end
```

Pseudocode P2. Code used to conduct the simulations on the effect of a global network signal on (non-existing) direct connectivity between seed and target revealed by ordinary and partial correlations. This simulation was repeated 50 times.

We present the results of this simulation Figure S2. It becomes obvious that a global network signal induces a strong connectivity between seed and target as measure by Ordinary Similarity analysis which can reach correlation values close to 1 when the influence of the glonbal network signal is extreme (40 times higher than the time course of seed and target). The Partial Similarity analysis shows that there is no direct connectivity between seed and target (Figure S2 A). To review the part of the simulation, which is more close to our actual data, we show in Figure S2 B an enlarged part of the results. As the maximally measured correlation coefficients are around r = 0.2 and the explained variance of the controlling variables lies around 30% the global network signal might have an influence of up to 60% in our real fMRI beta time series data.

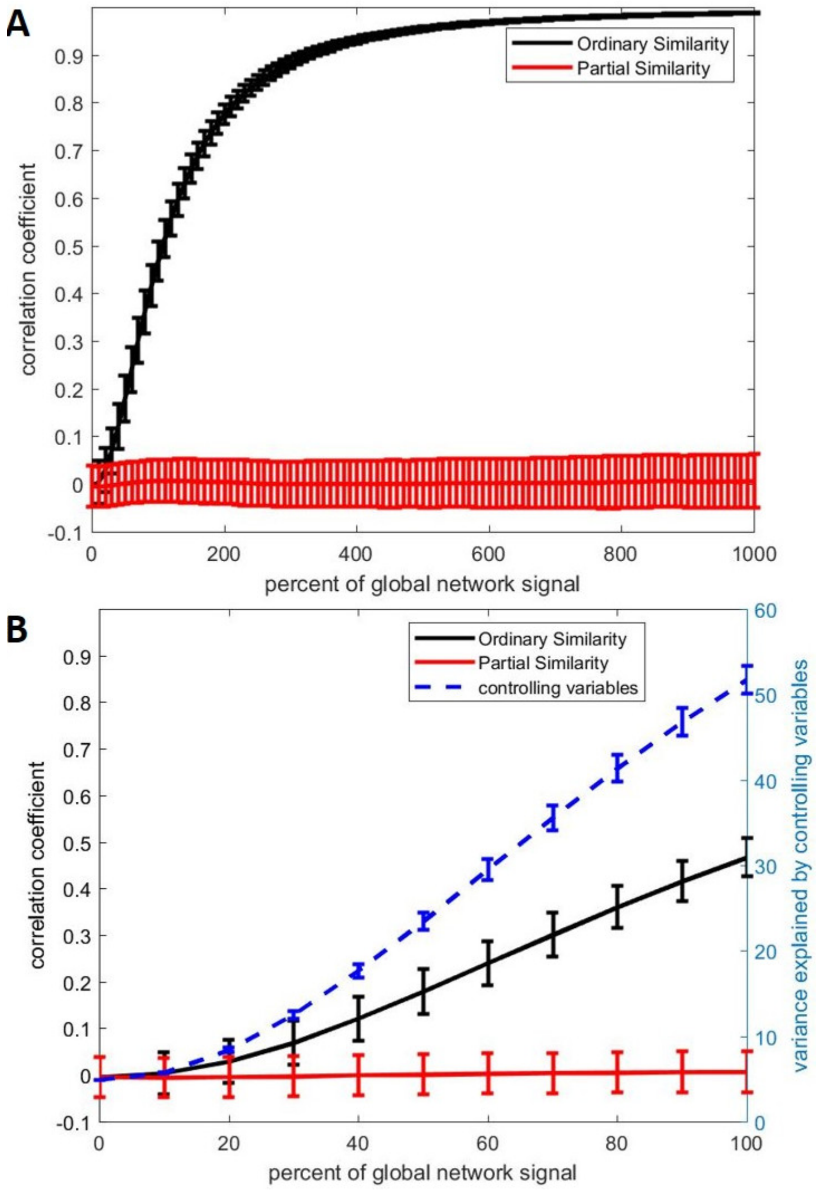

Figure S2. Results of the simulation of the Ordinary and Partial Similarity analysis of the simulated beta time series for 101 steps of 10% increment of global network influence while no direct connectivity between seed and target is simulated (**A**). For a closer view on the part of the simulation which is most close to our data we present increments from 0 to 100% in subfigure (**B**) and also include the variance explained by the first 15 principal components which are used as controlling variables for the Partial Similarity. Error bars indicate the standard deviation.

Simulation 2

We expanded this first simulation by introducing a signal common only for seed and target, representing a direct connectivity between them. While the global network signal again is mixed to all existing voxel, the common signal of seed and target is only mixed into seed and target voxel (see Pseudocode P2). In this second simulation, we can therefore simulate the complementary capability of Ordinal and Partial Similarity to disentangle the global network influence and the direct connectivity in the relation between seed and target region.

```
create a matrix with Gaussian noise in 100,000 voxel and 435 trials i.e. the beta time series
x_raw=random(10000,435);
create Gaussian noise used to induce correlations by the joint signal of the entire network
g=random(1,435);
create Gaussian noise used as common signal between source and target to simulate direct coomunication
d=random(1,435);
repeat for 11 mixing levels of the direct communication signal
for i=0 until 10
    repeat for 11 mixing levels of the global network signal
    for j=0 until 10
        add entire network signal with steps of 10% to all voxels
        x=x_raw+j/10*g;
        add direct communication signal with steps of 10% to seed and target
        x(0:1,:)= x(0:1,:)+i/10*d;
        calculate ordinary Spearman correlation between 1st and 2nd voxel, i.e. seed and target
        similarity(i)=spearmanCorr(x(0,:),x(1,:));
        calculate PCA for the remaining voxels excluding seed and target
        [b, C] = PCA(x(2:end,:));
        calculate partial Spearman correlation  controlling for 15 PCs
        partialSimilarity(i)=partialSpearmanCorr(x(0,:),x(1,:),C(0:14,:));
    end
end
```

Pseudocode P3. Code used to conduct the simulations on the effect of a global network signal on several levels of direct connectivity between seed and target revealed by Ordinary and Partial Similarity.

This second simulation shows that the Ordinary Similarity analysis reveals connectivity induced by a global network signal as well as a by a common signal between source and target. Nevertheless, a mixture cannot be disentangled while the Partial Similarity only measures the direct connectivity component of the mixed signals (Figure S3).

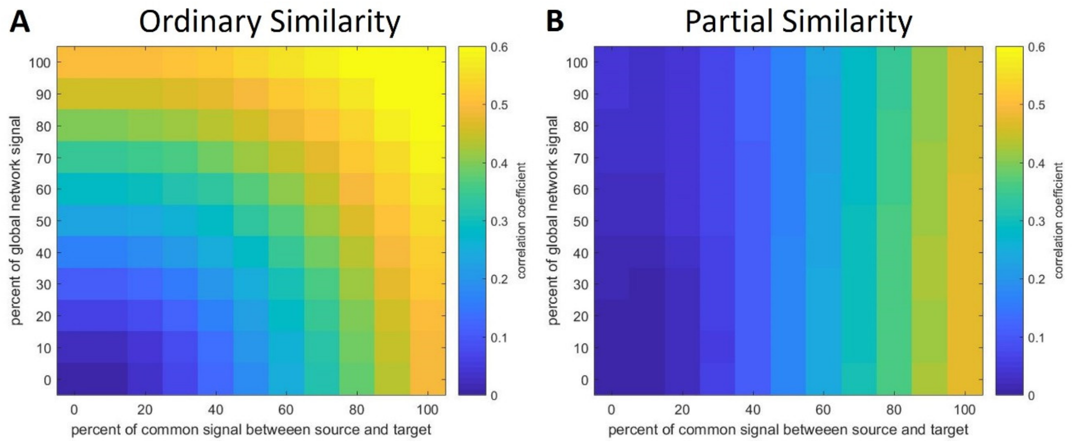

Figure S3. Results of the simulation of the Ordinary and Partial Similarity analysis of the simulated beta time series for 11 steps of 10% increment of global network influence while direct connectivity between seed and target is also simulated for 11 steps of 10% increment. While Ordinary Similarity (**A**) measures global connectivity as well as direct connectivity and a mixture of both, Partial Similarity (**B**) only reveals the direct connectivity component.

Simulation 3

In a third simulation, we simulate hidden correlations by adding the noise to half of the voxel but subtracting it from the other half. This simulation shows that a global network signal can even lead to negative correlations in the Ordinary Similarity analysis between a seed and a target region, which is not induced by analyzing the simulated data with Partial Similarity analysis (Figure S4). When global signal and direct connectivity have a similar magnitude the correlation coefficient for the Ordinary Similarity is near zero indicating no connectivity although seed and target possibly share common components. Here Partial Similarity is independent from the global influence and only reveals the direct component of the connectivity.

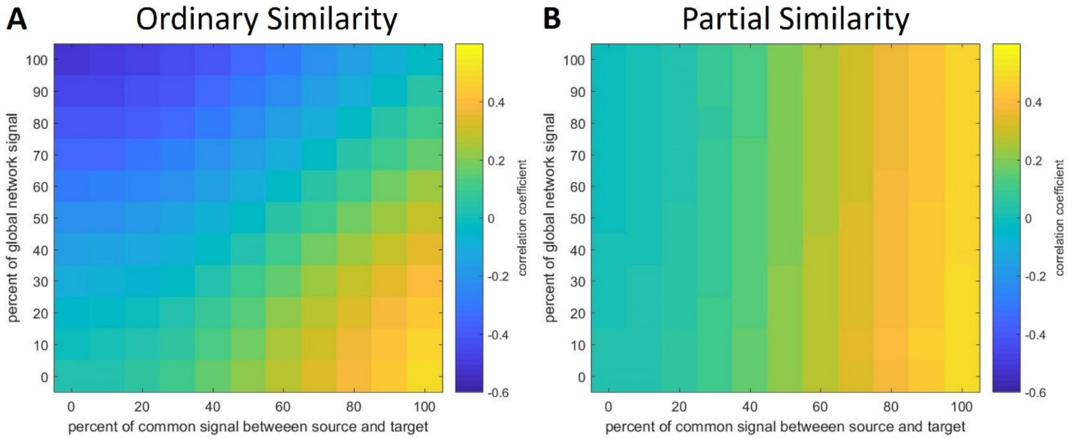

Figure S4. Results of the simulation of the Ordinary and Partial Similarity analysis of the simulated beta time series for 11 steps of 10% increment of global network influence. Here the global network signal has an additive effect on the seed and a negative effect on the target voxel. Again, we simulated the direct connectivity between seed and target for 11 steps of 10% increment. While Ordinary Similarity (**A**) analysis results in some cases even in negative correlations or nearly zero correlation, when global signal and direct connectivity have a comparable magnitude, Partial Similarity (**B**) only reveals the direct connectivity component.
